# Controversial issues in the management of hyperprolactinemia and prolactinomas – An overview by the Neuroendocrinology Department of the Brazilian Society of Endocrinology and Metabolism

**DOI:** 10.20945/2359-3997000000032

**Published:** 2018-03-23

**Authors:** Lucio Vilar, Julio Abucham, José Luciano Albuquerque, Luiz Antônio Araujo, Monalisa F. Azevedo, Cesar Luiz Boguszewski, Luiz Augusto Casulari, Malebranche B. C. Cunha, Mauro A. Czepielewski, Felipe H. G. Duarte, Manuel dos S. Faria, Monica R. Gadelha, Heraldo M. Garmes, Andrea Glezer, Maria Helane Gurgel, Raquel S. Jallad, Manoel Martins, Paulo A. C. Miranda, Renan M. Montenegro, Nina R. C. Musolino, Luciana A. Naves, Antônio Ribeiro-Oliveira, Cíntia M. S. Silva, Camila Viecceli, Marcello D. Bronstein

**Affiliations:** 1 Universidade Federal de Pernambuco Universidade Federal de Pernambuco Hospital das Clínicas Serviço de Endocrinologia Recife PE Brasil Serviço de Endocrinologia, Hospital das Clínicas, Universidade Federal de Pernambuco (UFPE), Recife, PE, Brasil; 2 Universidade Federal de São Paulo Universidade Federal de São Paulo Escola Paulista de Medicina Unidade de Neuroendócrino São Paulo SP Brasil Unidade de Neuroendócrino, Escola Paulista de Medicina, Universidade Federal de São Paulo (Unifesp/EPM), São Paulo, SP, Brasil; 3 Centro de Endocrinologia e Diabetes de Joinville Joinville SC Brasil Centro de Endocrinologia e Diabetes de Joinville (Endoville), Joinville, SC, Brasil; 4 Universidade de Brasília Universidade de Brasília Hospital Universitário de Brasília Serviço de Endocrinologia Brasília DF Brasil Serviço de Endocrinologia do Hospital Universitário de Brasília, Universidade de Brasília (UnB), Brasília, DF, Brasil; 5 Universidade Federal do Paraná Universidade Federal do Paraná Hospital de Clínicas Serviço de Endocrinologia e Metabologia Curitiba PR Brasil Serviço de Endocrinologia e Metabologia, Hospital de Clínicas, Universidade Federal do Paraná (SEMPR), Curitiba, PR, Brasil; 6 Universidade de São Paulo Universidade de São Paulo Faculdade de Medicina Divisão de Neurocirurgia Funcional, Instituto de Psiquiatria do Hospital das Clínicas São Paulo SP Brasil Divisão de Neurocirurgia Funcional, Instituto de Psiquiatria do Hospital das Clínicas, Faculdade de Medicina da Universidade de São Paulo (IPq-HC-FMUSP), São Paulo, SP, Brasil; 7 Universidade Federal do Rio Grande do Sul Universidade Federal do Rio Grande do Sul Faculdade de Medicina Serviço de Endocrinologia, Hospital de Clínicas de Porto Alegre, PPG Endocrinologia Porto Alegre RS Brasil Serviço de Endocrinologia, Hospital de Clínicas de Porto Alegre, PPG Endocrinologia, Faculdade de Medicina, Universidade Federal do Rio Grande do Sul (UFRGS), Porto Alegre, RS, Brasil; 8 Faculdade de Medicina da Universidade de São Paulo Hospital das Clínicas Serviço de Endocrinologia São Paulo SP Brasil Serviço de Endocrinologia, Hospital das Clínicas, Faculdade de Medicina da Universidade de São Paulo (FMUSP), São Paulo, SP, Brasil; 9 Universidade Federal do Maranhão Universidade Federal do Maranhão Hospital Universitário Presidente Dutra Serviço de Endocrinologia São Luís MA Brasil Serviço de Endocrinologia, Hospital Universitário Presidente Dutra, Universidade Federal do Maranhão (UFMA), São Luís, MA, Brasil; 10 Universidade Federal do Rio de Janeiro Universidade Federal do Rio de Janeiro Hospital Universitário Clementino Fraga Filho Serviço de Endocrinologia Rio de Janeiro RJ Brasil Serviço de Endocrinologia, Hospital Universitário Clementino Fraga Filho, Universidade Federal do Rio de Janeiro (HUCFF-UFRJ), Rio de Janeiro, RJ, Brasil; 11 Instituto Estadual do Cérebro Paulo Niemeyer Instituto Estadual do Cérebro Paulo Niemeyer Unidade de Neuroendocrinologia Rio de Janeiro RJ Brasil Unidade de Neuroendocrinologia, Instituto Estadual do Cérebro Paulo Niemeyer, Rio de Janeiro, RJ, Brasil; 12 Universidade Estadual de Campinas Universidade Estadual de Campinas Faculdade de Ciências Médicas Departamento de Clínica Médica Campinas SP Brasil Departamento de Clínica Médica, Faculdade de Ciências Médicas, Universidade Estadual de Campinas (FCM/Unicamp), Campinas, SP, Brasil; 13 Universidade Federal do Ceará Universidade Federal do Ceará Hospital Universitário Walter Cantídio Serviço de Endocrinologia Fortaleza CE Brasil Serviço de Endocrinologia, Hospital Universitário Walter Cantídio, Universidade Federal do Ceará (UFCE), Fortaleza, CE, Brasil; 14 Santa Casa de Belo Horizonte Serviço de Endocrinologia e Metabologia Belo Horizonte MG Brasil Serviço de Endocrinologia e Metabologia, Santa Casa de Belo Horizonte, Belo Horizonte, MG, Brasil; 15 Universidade Federal de Minas Gerais Universidade Federal de Minas Gerais Hospital das Clínicas Serviço de Endocrinologia Belo Horizonte MG Brasil Serviço de Endocrinologia, Hospital das Clínicas, Universidade Federal de Minas Gerais (UFMG), Belo Horizonte, MG, Brasil

**Keywords:** Hyperprolactinemia, prolactinomas, pseudoprolactinomas, macroprolactin, hook-effect, dopamine agonists, pituitary surgery, temozolomide

## Abstract

Prolactinomas are the most common pituitary adenomas (approximately 40% of cases), and they represent an important cause of hypogonadism and infertility in both sexes. The magnitude of prolactin (PRL) elevation can be useful in determining the etiology of hyperprolactinemia. Indeed, PRL levels > 250 ng/mL are highly suggestive of the presence of a prolactinoma. In contrast, most patients with stalk dysfunction, drug-induced hyperprolactinemia or systemic diseases present with PRL levels < 100 ng/mL. However, exceptions to these rules are not rare. On the other hand, among patients with macroprolactinomas (MACs), artificially low PRL levels may result from the so-called “hook effect”. Patients harboring cystic MACs may also present with a mild PRL elevation. The screening for macroprolactin is mostly indicated for asymptomatic patients and those with apparent idiopathic hyperprolactinemia. Dopamine agonists (DAs) are the treatment of choice for prolactinomas, particularly cabergoline, which is more effective and better tolerated than bromocriptine. After 2 years of successful treatment, DA withdrawal should be considered in all cases of microprolactinomas and in selected cases of MACs. In this publication, the goal of the Neuroendocrinology Department of the Brazilian Society of Endocrinology and Metabolism (SBEM) is to provide a review of the diagnosis and treatment of hyperprolactinemia and prolactinomas, emphasizing controversial issues regarding these topics. This review is based on data published in the literature and the authors' experience.

## INTRODUCTION

Hyperprolactinemia has multiple etiologies (Table 1) and is the most common endocrine disorder of the hypothalamic-pituitary axis (1–3). A prolactinoma is the most common cause of chronic hyperprolactinemia once pregnancy, primary hypothyroidism, and drugs that raise serum prolactin (PRL) levels have been ruled out (4–6).

**Table 1:** Causes of hyperprolactinemia

**Physiologic** Pregnancy; lactation; stress; sleep; coitus; exercise
**Pathologic** Systemic diseases – Primary hypothyroidism; adrenal insufficiency; renal insufficiency; cirrhosis; pseudocyesis; epileptic seizures Hypothalamic diseases – tumors (craniopharyngiomas, dysgerminomas, meningiomas, etc.); infiltrative disorders (histiocytosis, sarcoidosis, etc.), metastasis; cranial radiation; Rathke's cleft cysts, etc. Pituitary diseases – Prolactinomas; acromegaly; thyrotropinomas; Cushing's disease; infiltrative disorders; metastasis; lymphocytic hypophysitis; empty sella syndrome, etc. Stalk disorders – Hastitis; seccion; traumatic brain injury Neurogenic – Chest wall lesions (burns; breast surgery; thoracotomy; nipple rings; herpes zoster, etc.); spinal cord injury (cervical ependymoma; tabes dorsalis; extrinsic tumors, etc.), breast stimulation, etc. Idiopathic Ectopic prolactin production – Renal cell carcinoma; ovarian teratomas; gonadoblastoma; non-Hodgkin lymphoma, uterine cervical carcinoma; colorectal adenocarcinoma, etc.)
**Macroprolactinemia**
**Drug-induced (Table 3)**

Adapted from Ref. 1.

Prolactinomas are the most common hormone-secreting pituitary tumors, accounting for approximately 40% of all pituitary tumors (2,6) In adults, prolactinomas have an estimated prevalence of 60-100 per million population (7,8), and in a population from three different districts of Belgium, prolactinomas have been reported to represent 73.3% of all pituitary adenomas, with a higher prevalence in women (78.2%) (9). Between the age of 20 and 50 years, the ratio between women and men is estimated to be 10:1, whereas after the fifth decade of life, both genders are equally affected (10,11). Although prolactinomas are rare at the pediatric and adolescent ages, they account for approximately half of all pituitary adenomas in that population (12). PRL-secreting carcinomas are extremely rare (13).

The most characteristic signs and symptoms found in patients with hyperprolactinemia are those related to hypogonadotropic hypogonadism and galactorrhea (1,3,7). Increased PRL levels decrease gonadotropin pulsatile secretion through inhibition of hypothalamic GnRH release (14). In addition, there may be direct effects of hyperprolactinemia on testes and ovaries. Hypogonadism can cause menstrual irregularity and amenorrhea in women, sexual dysfunction, infertility, and loss of bone mineral mass in both genders (15,16). Hyperprolactinemia can also reduce the libido independently of testosterone levels (17). In patients harboring macroprolactinomas, tumor mass effect symptoms, such as headache, visual changes, and, more rarely, cerebrospinal fluid (CSF) rhinorrhea, hydrocephalus and seizures, can also occur (1–3). Hypopituitarism beyond hypogonadism can occur if there is compression of the pituitary stalk or destruction of normal pituitary tissue (3,6,7). It is worth commenting that some women present with non-puerperal galactorrhea in the presence of regular menstrual cycles and normal PRL levels (18,19). This so-called “idiopathic galactorrhea” is estimated to be present in up to 40-50% of all women with non-puerperal galactorrhea (19,20). In contrast, the finding of galactorrhea in men is highly suggestive of a prolactinoma (2,18).

In this publication, the goal of the Neuroendocrinology Department of the Brazilian Society of Endocrinology and Metabolism (SBEM) is to provide a review on the diagnosis and treatment of hyperprolactinemia and prolactinomas, emphasizing controversial issues regarding these topics.

## PROLACTIN SERUM ISOFORMS

The PRL size is heterogeneous in terms of circulating molecular forms. The predominant form in healthy subjects and in patients with prolactinomas is monomeric PRL (molecular weight of 23 kDa), while dimeric or big PRL (45-60 kDa) and big-big PRL or macroprolactin (150-170 kDa) correspond to less than 20% of the total PRL (20,21). When the serum of a patient with hyperprolactinemia contains mostly macroprolactin, the condition is termed macroprolactinemia (22,23). In up to 90% of cases, macroprolactin is composed of a complex formed by an IgG and monomeric PRL (2,24–29).

## DIAGNOSTIC EVALUATION

For the correct identification of the etiology of hyperprolactinemia, some parameters must be taken into account: medical history, physical examination, clinical features, laboratory findings (especially PRL serum levels), and imaging studies of the pituitary and sella turcica (1,3,5). Furthermore, the screening for macroprolactinemia should often be considered, particularly in cases of asymptomatic hyperprolactinemia (3–5).

In addition to PRL determination, TSH, free T^4^, and creatinine levels should be obtained to rule out secondary causes of hyperprolactinemia (1,3,6). Moreover, acromegaly must be investigated by measuring IGF-1 in all patients with a macroadenoma, even when there are no manifestations of this disease (30). Finally, β-hCG measurement is mandatory in any woman of childbearing age with amenorrhea (1,3).

## 1. CONTROVERSIAL ISSUES REGARDING DIAGNOSIS

### 1.1. Environmental influences on PRL secretion

Prolactin is secreted in a pulsatile manner, and serum levels can vary greatly throughout the day, with higher levels during sleep, a morning peak and a gradual decline after awakening, but without a typical circadian rhythm. Under normal conditions, ~50% of the total daily production of PRL occurs during the sleep period. Thus, samples should be collected up to 3 hours after awakening, preferably while the patient is fasted (1,4).

Stress from any source, whether psychological, induced by exercise or due to other acute illness, leads to the physiological elevation of PRL levels (2,3,5). However, supine rest is not necessary prior to sampling, contrary to what was believed in the past (31). Venipuncture stress may cause an elevation in the PRL level, but it is usually mild (< 40-60 ng/mL) (32). The same is true for breast stimulation (1,2,33). Moreover, as PRL is secreted episodically, its levels measured during the day may possibly be beyond the upper limit of normality for a particular laboratory in healthy individuals (1,3). Therefore, an elevated PRL level should be confirmed at least once (33) unless the PRL levels are clearly elevated (> 80-100 ng/mL) (1). Nevertheless, according to the guidelines of the Endocrine Society, a single PRL level above the upper limit of normal confirms the diagnosis of hyperprolactinemia, as long as the serum sample was obtained without excessive venipuncture stress (4).

**COMMENT 1:** As PRL is secreted in a pulsatile manner and as venipuncture stress can increase PRL levels, we suggest that an elevated PRL level should be confirmed at least once, unless the PRL levels are clearly elevated (> 80-100 ng/mL).

**COMMENT 2:** Vigorous exercise and nipple stimulation should be avoided for at least 30 minutes before checking PRL levels as they may result in PRL elevation.

### 1.2 Accuracy of prolactin levels

The magnitude of PRL elevation can be useful in determining the etiology of hyperprolactinemia because the highest values are observed in patients with prolactinomas (1–5,33,34). For example, levels > 250 ng/mL are highly suggestive of the presence of a prolactinoma (3–5), although they may occasionally be found in other conditions (1,34), as commented on below. In contrast, most patients with stalk dysfunction (pseudoprolactinomas), drug-induced hyperprolactinemia or systemic diseases present with PRL levels < 100 ng/mL (1,4,34). However, exceptions to these rules are not rare (1,34).

In patients with prolactinomas, circulating PRL levels usually parallel the tumor size (1,4,7). Indeed, microprolactinomas (MIC) (diameter < 10 mm) usually result in PRL levels of 100-200 ng/mL, but not infrequently, they may be < 100 ng/mL, and occasionally reach 500 ng/mL or more (1,33,34). Macroprolactinomas (MACs) (diameter ≥ 10 mm) are typically associated with PRL values > 250 ng/mL (4–7). In the vast majority of patients with giant prolactinomas (maximum diameter ≥ 4 cm), PRL levels will be > 1000 ng/mL (35,36). On the other hand, artificially low PRL levels may result from the so-called “hook effect”, which should be considered in all cases of large (≥ 3 cm) pituitary macroadenomas associated with normal or mildly elevated PRL levels (< 200 ng/mL) (1,37–39), as commented on below. Patients harboring cystic MACs may also present with mild PRL elevation (1–3).

**Figure 1:**
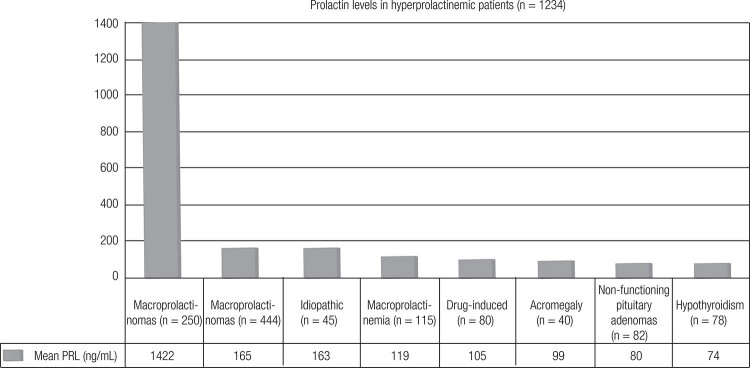
PRL levels according to the etiology of the hyperprolactinemia in the Brazilian multicenter study on hyperprolactinemia (Adapted from Ref. 34).

The Brazilian Multicenter Study on Hyperprolactinemia (BMSH) analyzed 1234 patients whose results are presented in Figure 1 and Table 2 (34). In this study, only patients harboring prolactinomas presented with PRL values ≥ 500 ng/nL (34).

**Table 2:** Prolactin levels (ng/mL) according to the etiology of the hyperprolactinemia in the Brazilian Multicenter Study on Hyperprolactinemia

Etiology	N (%)	Mean PRL (range) (ng/mL)
Macroprolactinomas	250 (20.2)	1422.9 ± 3134.7 (108-21,200)
Microprolactinomas	444 (36)	165.6 ± 255.1 (32-525)
Idiopathic	45 (3.6)	163.9 ± 81.8 (46-328)
Macroprolactinemia	115 (9.3)	119.5 ± 112.9 (32.5-404)
Drug-induced	180 (14.6)	105.1 ± 73.2 (28-380)
Acromegaly	40 (3.2)	99.3 ± 57.4 (28-275)
NFPA	82 (6.6)	80.9 ± 54.5 (28-490)
Primary hypothyroidism	78 (6.3)	74.6 ± 42.4 (30-253)

NFPA: Non-functioning pituitary adenomas. Adapted from Ref. 34.

#### 1.2.1 How do PRL levels behave in cases of pseudoprolactinomas?

In patients with “pseudoprolactinomas”, whose main etiology isa nonfunctioning pituitary adenoma (NFPA), hyperprolactinemia results from compression of the pituitary stalk (2,40). In that situation, this so-called disconnection hyperprolactinemia is thought to result from loss of the inhibitory effect of dopamine on PRL secretion (19). NFPAs represent the principal differential diagnosis of macroprolactinomas, as they require distinct treatments and have a distinct natural history and prognosis (1,40). The term pseudoprolactinomas also includes other conditions such as craniopharyngiomas, Rathke's cleft cysts, sarcoidosis, Langerhans-cell histiocytosis and metastasis (19,40).

Based on a large series of histologically confirmed cases (n = 226) with NFPA, serum PRL > 2000 mIU/L (> 95 ng/mL) is almost never (< 2%) encountered in these patients (41). Accordingly, in a recent study, among 64 patients with immunohistochemically confirmed NFPAs, PRL levels ranged from 33 to 250 ng/mL (~80% < 100 ng/mL) (42) By contrast, in BMSH, among 82 patients with NFPA, PRL levels ranged from 28 to 490 ng/mL (< 100 ng/mL in 82%); however, not all patients had been submitted to immunohistochemical evaluation (Table 3) (34).

**Table 3:** Drug-induced hyperprolactinemia

**Antipsychotics** Typical – Phenothiazines; butirophenones; thyoxanthenes Atypical – Risperidone; molindone; amisulpride; quetiapine; olanzapine
**Antidepressants** Tricyclics – Amitriptyline; desipramine; clomipramine MAO inhibitors – Pargyline; clorgyline SSRIs – Fluoxetine; citalopram; paroxetine
**Antihypertensive drugs** Verapamil; α-methyldopa; reserpine; labetolol
**Anticonvulsivants** Phenytoin
**Prokinetic agents** Metoclopramide; domperidone; bromopride
**Others** Estrogens; anesthetics; cimetidine; ranitidine; opiates; methadone; morphine; apomorphine; heroin; cocaine; marijuana; alcohol; sibutramine, etc.

MAO: monoamine oxidase; SSRIs: selective serotonin re-uptake inhibitors. Adapted from Refs. 1, 2, and 3.

**COMMENT 3:** In cases of non-functioning pituitary adenomas, hyperprolactinemia results from stalk compression, and thus, prolactin (PRL) levels are modestly elevated (< 100 ng/mL) in the great majority of cases. Values > 250 ng/mL are exceedingly rare. By contrast, in patients with macroprolactinomas, PRL levels are usually > 250 ng/mL, and not infrequently, they exceed 1000 ng/mL. However, PRL levels may be misleadingly low due to the hook effect or in patients with cystic macroprolactinomas.

#### 1.2.2 How do PRL levels behave in cases of drug-induced hyperprolactinemia?

The most common cause of non-physiological hyperprolactinemia is the use of drugs, which act through different mechanisms: increased transcription of the PRL gene (*estrogens*), antagonism of the dopamine receptor (*risperidone*, *haloperidol*, *metoclopramide, domperidone, sulpiride, etc.*), dopamine depletion (*reserpine, methyldopa*), inhibition of hypothalamic dopamine production (*verapamil, heroin, morphine, enkephalin analogs, etc.*), inhibition of dopamine reuptake (*tricyclic antidepressants, cocaine, amphetamine, monoamine oxidase inhibitors*), inhibition of serotonin reuptake (opiates, fenfluramine, fluoxetine, sibutramine), etc. (1,2,42–47) (Table 3).

In the Brazilian Multicenter Study on Hyperprolactinemia (BMSH), antidepressants and neuroleptics (in monotherapy or in combination) were the culprits in a large majority of the cases (82.2%) (34). Among antipsychotics, the most frequently involved drugs were haloperidol, phenothiazines, and risperidone, while tricyclic drugs were the main representants among the antidepressants (34). Other studies found the following rates of hyperprolactinemia associated with each therapeutic drug class: 31% for neuroleptics, 28% for neuroleptic-like drugs, 26% for antidepressants, 5% for H2-receptor antagonists, and 10% for other drugs (45). In one group of 106 patients receiving antipsychotics, hyperprolactinemia was present in 81%, 35%, 29%, and 38% of patients taking risperidone, olanzapine, ziprasidone, and typical antipsychotics, respectively (46).

The newer atypical antipsychotics (AAPs) are characterized by increased antipsychotic efficacy and fewer neurological and endocrine related side-effects compared to classical antipsychotic drugs (45,46). With the exception of risperidone, amisulpride and molindone, which are often associated with high PRL levels (45), most of the AAPs elicit a poor hyperprolactinemic response or no hyperprolactinemia at all (43,45,46). Furthermore, the use of drugs such as quetiapine and aripiprazole (a dopamine partial agonist) was shown to be associated with resolution of the hyperprolactinemia induced by other AAPs (48). Moreover, decreased PRL levels were also reported when aripiprazole was used as adjunct therapy to risperidone (49).

Antidepressants induce hyperprolactinemia in a small proportion of patients, but they rarely elevate PRL to a significant degree (46). Among 80 patients treated with fluoxetine, only 10 (12.5% developed hyperprolactinemia, with 38 ng/mL being the highest PRL level (50). Atypical antidepressants, including bupropion and mirtazapine, appear to have no effect on PRL levels (44,47).

Although PRL elevation is usually mild (25-100 ng/mL) in cases of drug-induced hyperprolactinemia, it is also highly variable. Indeed, metoclopramide, risperidone, and phenothiazines can lead to prolactin levels > 200 ng/mL (1,43–46). Among 180 cases enrolled in the BMSH, most (64%) presented with PRL levels < 100 ng/mL, but in 5%, they exceeded 250 ng/mL (range, 28-380; mean, 105.1 ± 73.2) (Table 3) (34). Interestingly, PRL levels of 720 ng/mL were recently reported in a young lady who had been treated with domperidone for 3 months. Following domperidone discontinuation, PRL fell to the normal range (51).

**COMMENT 4:** Although drug-induced hyperprolactinemia is usually associated with PRL levels < 100 ng/mL, they are largely variable and may overlap those found in patients with prolactinomas.

#### 1.2.3 How do the hook effect and linearity issues in PRL assays impact our practice?

**Figure 2:**
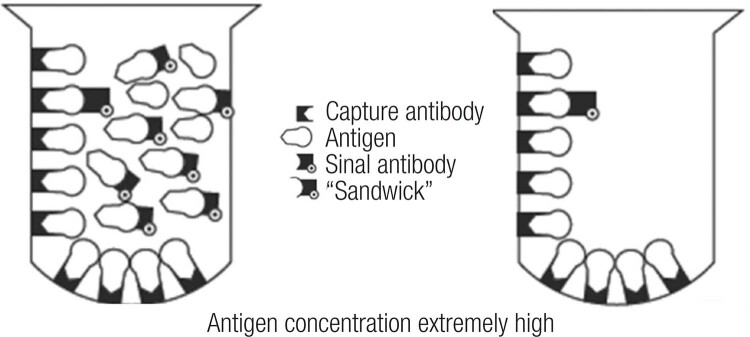
Schematic depiction of “hook effect.” *Left*, Extremely high antigen concentrations saturate both capture and signal antibodies and prevent “sandwich” formation. *Right*, When liquid phase is discarded, most of the antigen is lost with the signal antibody; thus, antigen concentration is measured as low (Adapted from Ref. 39).

Immunometric assays have greatly improved the sensitivity of PRL and other hormone measurements. They are usually performed through capture antibodies that are immobilized in a solid phase, and a second antibodyis labeled a signal generator. These antibodies bind to different epitopes of the PRL to be quantified, thus forming a “sandwich” test using either a fluorescent or chemiluminescent marker. The relative antigen-to-antibody proportion influences its interaction and may compromise the appropriate formation of the immunocomplexes. Thus, extremely high concentrations of PRL can simultaneously saturate both the capture and the labeled antibody when only a few PRL molecules are actually bound in a sandwich complex to be quantified by the test. In that situation, most of the PRL molecules are bound to just one antibody instead and are subsequently washed away (Figure 2). Therefore, falsely low results are reported, and the correct result for the PRL concentration is much higher than reported. This artifact is called a high-dose hook effect, also known as the prozone phenomenon, and the reported results are usually within or, more often, slightly above the manufacturer's reference range (52,53).

The hook effect differs between the different assay systems used in clinical practice. Dilution of the sample at 1:100 is the test of choice to unmask this hook effect (1,4). Indeed, this step will result in a dramatic rise in PRL levels if the patient has a macroprolactinoma, remaining low in cases of non-functioning pituitary adenomas (1,4,37,38).

Physicians should keep in mind that laboratories cannot dilute all samples on a routine basis to rule out the hook effect. Thus, it is extremely important that they are aware of the phenomenon so they do not forget to order dilution for all PRL samples suspected to be overconcentrated. In clinical practice, this means that dilutions must be ordered for PRL measurements in all patients with macroadenomas ³ 3 cm and initial PRL levels < 200 ng/mL, even if the PRL levels are normal (1,2,38). However, it is relevant to mention that dilutions ordered by physicians are performed at the discretion of laboratory workers. This means that, according to the reportable reference range of an assay and its reported levels of hook effect by the respective manufacturer, dilutions may occasionally start at 1:10. For instance, considering an assay in which, according to its manufacturer, the hook effect is not supposed to be induced up to a PRL value of 20,680 ng/mL, this theoretically means that a starting dilution of 1:10 should be sufficient enough to detect the phenomenon, as it is very improbable to have a PRL value higher than 200,680 ng/mL (53). In this regard, it is noteworthy that unnecessary dilutions usually cause a loss of accuracy in measurements. Fortunately, with the newer assays, extremely high levels of PRL are usually necessary to hook the assay, and this fact has dramatically decreased the incidence of this phenomenon (53,54).

Interestingly, the hook effect has often been confused with assay linearity problems by clinicians (54). In a given assay with a reportable PRL ranging from 0.25-200 ng/mL according to the manufacturer, PRL samples coming from patients with untreated macroprolactinomas may often fall out of this reportable range, even when an automatic dilution of 1:10 is superimposed. In this case, it is common that the laboratory releases a result of “> 2000 ng/mL”, and it seems that they are not aware of the importance of exact quantification of the result. Therefore, clinicians must refrain from starting treatment until the laboratory re-assays the sample at further dilutions, even if it has to be performed manually, and up to the point that the linear range of the assay is reached. Otherwise, the observed effect of a treatment may be misled due to an inexact reported measurement (53,54).

**COMMENT 5:** The hook effect should be considered in every patient presenting with a large (≥ 3 cm) pituitary macroadenoma and prolactin levels within the normal range or only modestly elevated.

### 1.3 Macroprolactinemia screening: routinely or just in asymptomatic individuals?

Macroprolactinemia is a condition where more than 60% of circulating PRL is made up of macroprolactin (1,29). In most of the *in vitro* studies, macroprolactin was shown to display low biological activity (28,29,55). This is corroborated by the finding that in most series with macroprolactinemia, individuals are pauci- or asymptomatic (56,57), with no need to perform sellar imaging (58) or specific treatments (58,59). Others argued that the binding of PRL to their receptor could be blocked by modification of the tertiary structure of the original molecule (60).

However, there are individuals who, despite increased macroprolactin, also present with high levels of monomeric PRL, leading to “true” hyperprolactinemia with clinical symptoms and the need foran etiologic diagnosis for the proper management of hyperprolactinemia (59). Moreover, the presence of symptoms could result from the concomitance of macroprolactinemia with other conditions, such as polycystic ovary syndrome (61), idiopathic galactorrhea (1,2), or psychogenic erectile dysfunction (62).

Assaying serum PRL before and after precipitation with polyethylene glycol (PEG) is the most used method for screening macroprolactinemias due to its low cost and easy workability (63). Theoretically, macroprolactin is precipitated with PEG, and only monomeric PRL will be recovered in the supernatant. However, some monomeric PRL also suffers precipitation, hence the need for the standardization of normal monomeric PRL values after PEG precipitation (58,60). Recoveries < 40% are indicative of the predominance of macroprolactin, whereas recoveries > 60% point to the diagnosis of monomeric hyperprolactinemia (56,58). Overall, PEG precipitation enables the correct diagnosis of macroprolactinemia in at least 80% of cases (56,63). The gold standard diagnostic test is the separation of isoforms by gel filtration, which correlates well with the PEG precipitation and is the only way of assessment when screening with PEG is inconclusive (58,63). However, it is an expensive and time-consuming method that cannot be used routinely.

It is noteworthy that different assays recognize macroprolactin differently (64). It has been demonstrated that some of the new assays show lower cross-reactivity with macroprolactin; however, the number of samples defined as macroprolactin is still significant (65).

Hyperprolactinemia related to macroprolactin may be due to its lower renal clearance, longer half-life and lower capability to activate hypothalamic dopaminergic tone, which negatively regulates the secretion of pituitary prolactin (5). The frequency of macroprolactinemia in the general population was shown to be 0.2% in women from Scandinavia (66) and 3.7% in a total of 1330 Japanese hospital workers of both genders (67), whereas among hyperprolactinemic individuals, it ranged from 8 to 42%, with a mean of 19.6%, in 8 European series (56,68–75). The study population may explain the variation in the frequency of macroprolactinemia in hyperprolactinemic individuals. As an example, two Brazilian studies have shown frequencies of 16.5% in 115 consecutive patients with hyperprolactinemia (76) and 46% in 113 cases from a reference laboratory (58). This high frequency of macroprolactinemia becomes an important issue in clinical practice: what is the probability with an additional assessment and treatment in an individual presenting with macroprolactinemia? Should all individuals with hyperprolactinemia be actively investigated for the presence of macroprolactinemia?

**Figure 3:**
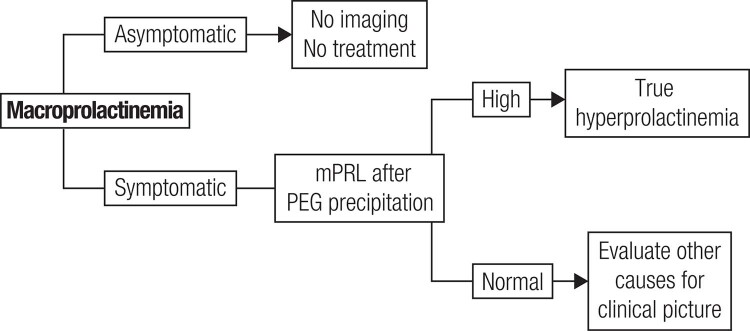
Flowchart to the management of patients with macroprolactinemia (mPRL: monomeric prolactin) (Adapted from Ref. 6).

The request for serum PRL assessment occurs in two scenarios. At first, there are complaints related to hyperprolactinemia, such as galactorrhea, hypogonadism and infertility, leading to serum PRL measurement. If laboratory tests confirm the clinical suspicion of monomeric hyperprolactinemia, macroprolactinemia screening is not indicated, and it is recommended to proceed to the usual investigation of physiological, pharmacological and pathological causes of hyperprolactinemia for proper handling of the case. In the second scenario, serum PRL evaluation is requested in the absence of complaints related to hyperprolactinemia. In this situation, facing hyperprolactinemia in an asymptomatic individual, macroprolactinemia screening is always indicated. If positive and monomeric PRL levels are normal, it should guide the patient that there is no need for further investigation, follow-up or treatment, due to the benign nature of the condition. If the screening with PEG is inconclusive, one may proceed to gel-filtration chromatography, or if the latter is unavailable, be guided by the clinical picture. If the macroprolactin result is negative, investigate hyperprolactinemia as usual. A flowchart for the management of macroprolactinemic individuals is proposed in Figure 3.

Some authors advocate routine macroprolactin screening as a cost-effective procedure (56,76–78), and others allow screening to rule out macroprolactinemia and investigate other conditions that justify symptoms (79). In a study conducted in a Brazilian reference laboratory, there was more cost in searching for individuals with true hyperprolactinemia, but screening macroprolactinemia did not prevent investigation and inappropriate treatment, pointing to the need for the dissemination of medical knowledge about macroprolactinemia (80). By contrast, according to some studies, the detection of macroprolactin may change the initial diagnosis in a significant proportion of patients. Indeed, in three series (73,74,81), macroprolactinemia was encountered in 25 to 68.3% (mean, 42.3%) of patients with apparent idiopathic hyperprolactinemia (IH). Moreover, the diagnosis of PRL-secreting pituitary microadenoma shifted to non-secreting pituitary microadenoma in 10 of 49 patients (20%) reported by Donadio and cols. (73). Thus, macroprolactinemia may occasionally represent a relevant cause of misdiagnosis, unnecessary investigation and inappropriate treatment (73,74,81). Conversely, PRL should never be measured in asymptomatic patients, in order to avoid the unnecessary detection of macroprolactinemia cases (1,5).

Concerning the natural history of macroprolactinemia, macroprolactinemic subjects usually display persistent macroprolactinemia without the development of raised free PRL (82). However, during follow-up, hyperprolactinemia may develop in macroprolactinemic subjects who were initially normoprolactinemic along with an increase in anti-PRL autoantibody titers (82).

Overall, symptoms related to hyperprolactinemia (galactorrhea, menstrual disorders and sexual dysfunction) have been reported in up to 45% of patients with macroprolactinemia (56,58,69,70,73,74,76,83,84). As mentioned, this would mostly result from the concomitance with monomeric hyperprolactinemia (74,75,85) or other disorders, such as polycystic ovary syndrome (1,61,62). Of note, the finding of both galactorrhea and menstrual disorders is rare in macroprolactinemia (83,84).

In most patients with macroprolactinemia, PRL levels are < 100 ng/mL, but they are highly variable: from 20-663 ng/mL (mean, 61 ± 66; < 100 ng/mL in approximately 91% of cases) (83), to 119.5 ± 112.9 (range, 32.5-404; < 100 mg/L in 74%) among 115 patients in the BMSH (33). In most studies, PRL levels were lower in macroprolactinemic patients than in those with monomeric hyperprolactinemia, but there was a great overlap between groups (34,56,63,69,83). Moreover, MRI abnormalities (e.g., macroadenomas and, mostly, microadenomas or an empty sella) may be found in approximately 20-25% of macroprolactinemic patients (69,70,73,74,84).

**COMMENT 6:** Macroprolactinemia is, in most cases, a laboratory diagnostic pitfall, with a mean frequency of ~20% among hyperprolactinemic subjects. Clinical, radiological and laboratory features cannot be used reliably to differentiatemonomeric hyperprolactinemia from macroprolactinemia. The screening for macroprolactin is mostly indicated for asymptomatic hyperprolactinemic patients, subjects with apparent idiopathic hyperprolactinemia, and any patient without an obvious cause for the hyperprolactinemia.

## TREATMENT OF HYPERPROLACTINEMIA AND PROLACTINOMAS

The ideal treatment of hyperprolactinemia depends on its etiology and may include, for instance, L-thyroxine replacement in patients with primary hypothyroidism, dopamine agonists (DAs) for prolactinomas, and drug withdrawal in cases of drug-induced hyperprolactinemia (2–7). By contrast, macroprolactinemia does not need to be treated (5,24,25).

**Figure 4:**
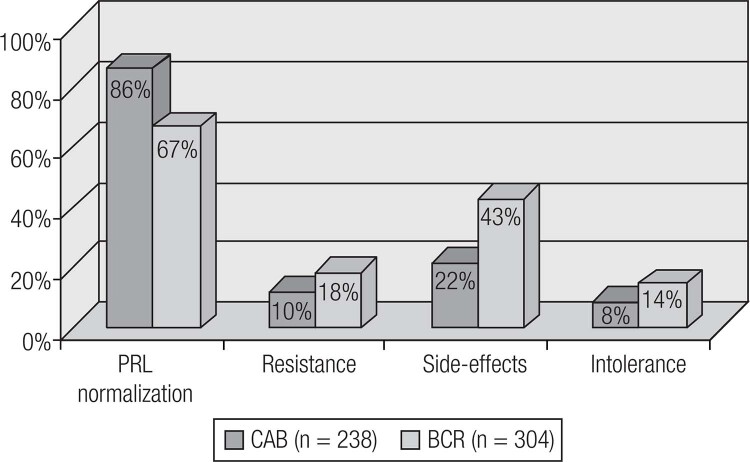
Comparison of cabergoline (CAB) and bromocriptine (BCR), concerning efficacy and tolerability (Adapted from Ref. 34).

**Figure 5:**
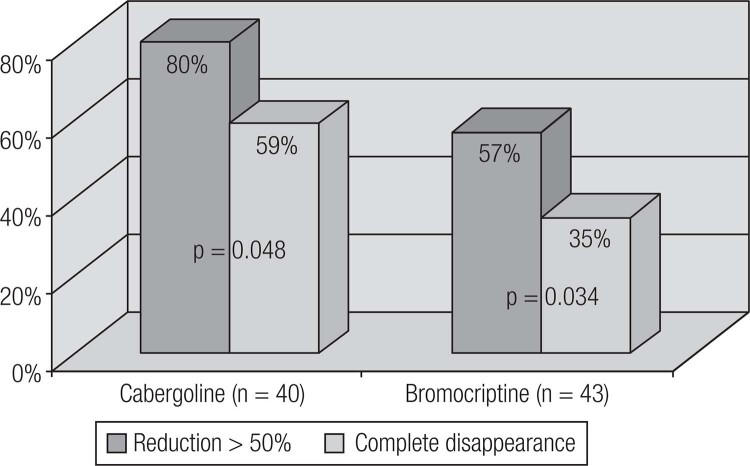
Comparative efficacy of CAB and BCR in inducing tumor shrinkage in naïve patients with macroprolactinomas (Adapted from Ref. 34).

**Figure 6:**
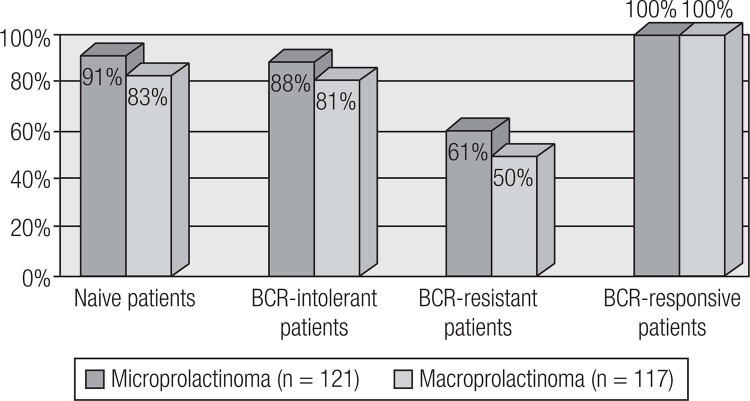
Efficacy of cabergoline on the normalization of PRL levels in 238 patients with prolactinomas. BCR: bromocriptine (Adapted from Ref. 34).

**Figure 7:**
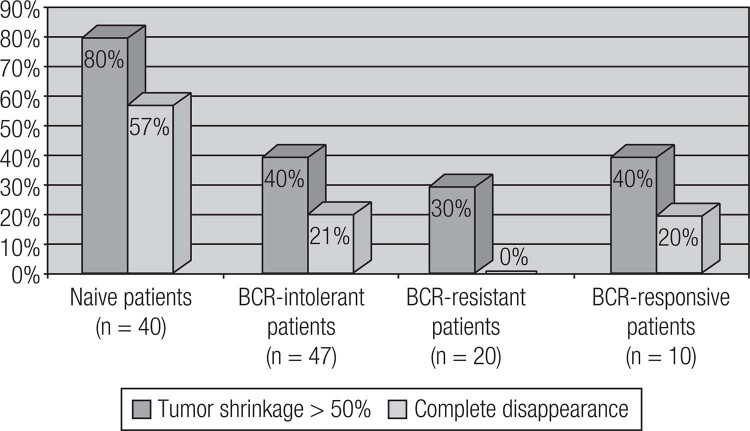
Concerning macroprolactinoma shrinkage, cabergoline is effective not only in naive patients but also in those previoiusly treated with bromocriptine (BCR) (Adapted from Ref. 34).

Current available therapeutic options for prolactinomas include surgery, pituitary radiation therapy and pharmacotherapy with dopamine agonists (DAs) (19). DAs are the gold standard treatment for prolactinomas, as their use controls hormonal secretion and tumor growth in approximately 80% of cases (8). Among DAs, bromocriptine (BRC) and cabergoline (CAB), both of which are ergot derivatives, are the most commonly used worldwide. Quinagolide is available in some European countries. Cabergoline (CAB), a specific agonist of the dopamine receptor type 2 (D^2^R), is the first choice because of its better tolerability and greater efficacy in inducing PRL normalization and tumor shrinkage (Figures 4 and 5) (Table 4) (19,86–89). Bromocriptine use leads to normal serum PRL levels in 80% of microprolactinomas and 70% of macroprolactinomas, whereas with CAB, this goal is achieved in 85% of patients (3,86–88). CAB effectiveness is higher in naïve patients, but the drug is also very effective in patients with intolerance or resistance to BCR (Figures 6 and 7) (34,88). The better performance of CAB probably results from its better tolerance and higher affinity for D_2_R (10,19,88).

**Table 4:** Comparative efficacy of cabergoline (CAB) and bromocriptine (BCR) among patients with macroprolactinomas from the Brazilian Multicenter Study on Hyperprolactinemia

Outcome	CAB (n = 117)	BCR (n = 133)	p-value
PRL normalization	77.8%	59.4%	0.042
Tumor reduction > 50%	80%	58.7%	0.048
Complete tumor disappearance	57.5%	34.7%	0.034

Adapted from Ref. 34.

## 2. CONTROVERSIAL ISSUES REGARDING TREATMENT

In this topic, we will cover challenging or controversial aspects related to the treatment of prolactinomas and the management of psychotropic*-*induced hyperprolactinemia.

### 2.1 How to manage the resistance to dopamine agonists?

Different arbitrary concepts have been proposed for the definition of resistance to dopamine agonist (DA) therapy (2,4,88–91). Currently many experts have adopted the definition suggested by Molitch, which includes failure to normalize PRL levels and to decrease macroprolactinoma size by ≥ 50% with maximal conventional doses of medication (7.5 mg/day of bromocriptine or 2.0 mg/week of cabergoline) (89,90). Bromocriptine (BCR) fails to normalize prolactin levels in approximately one-quarter of patients; cabergoline (CAB), in 10-15%. BCR fails to decrease prolactinoma size by at least 50% in approximately one-third of the patients; CAB, in 10-15% (34,88–91).

#### Pathogenetic mechanisms

There are a number of potential mechanisms to explainresistance to DAs. Drug absorption or drug affinity to D^2^R are not involved (90). DA resistance is rather associated with a decrease in D^2^R gene transcription, resulting in a 4-fold decrease in the number of D^2^Rs on the cell membrane (92,93). Moreover, there is a decrease in the G protein that couples the D^2^R to adenylyl cyclase, further decreasing the ability of dopamine to inhibit PRL secretion (90).

Patients who initially respond to a DA may rarely become resistant to these drugs at a later point in time (90). Most commonly, this is due to noncompliance. Rarely, there may be malignant transformation of a prolactinoma (94). In some cases, the development of DA resistance is due to the concomitant use of hormone replacement therapy with estrogen or testosterone (95).

#### Treatment

The approaches for patients with resistance to DA therapy include (1) switching to another DA; (2) raising the dose of the DA beyond conventional doses if the patient continues to respond and tolerate; (3) surgical tumor resection; (4) radiotherapy; and (5) experimental treatments with other drugs (34,88,90,96,97).

#### Switching to another DA

Most of the data regarding switching dopamine agonists in resistant patients address switching from BCR to CAB. CAB is effective in normalizing PRL levels in approximately 50-80% of patients resistant to BCR, and up to 70% respond with some tumor size change (30,90,96,97). It is not clear why CAB should be so effective in patients resistant to BCR, but this may be due to cabergoline's possessing a higher affinity for dopamine binding sites, a longer time occupying the receptor, and a slower elimination from the pituitary (90,97). By contrast, the response to BCR in a patient resistant to CAB is much more rare and has only been reported twice (90,98).

#### Raising the dose of the DA beyond conventional doses

As long as there is a continued response and no adverse effects from higher doses, there is no reason not to continue to increase the CAB dose (90). There is, however, a concern regarding the risk of cardiac valvulopathy induced by high doses of CAB (see below) (88,90).

Using an individualized, stepwise approach of dose titration of CAB, Ono and cols. (99) documented that 25 of 26 patients (96.1%) considered to have DA resistance, achieved normalization of the PRL levels within 12 months, with a mean dose of CAB of 5.2 ± 0.6 mg per week (range 3-12 mg/week). The rate of PRL normalization gradually increased to 35, 73, and 89% at 3, 6, and 9 mg/week, respectively, finally reaching 96% at the highest dose of 12 mg/week (99). DiSarno and cols. (100) found that doses > 2.0 mg/week (up to 7 mg/week) of CAB werestill unable to normalize PRL levels in 18% of patients with macroadenomas and 10% of those with microadenomas. More recently, Vilar and cols. (101) prospectively evaluated the management of 25 prolactinomas refractory to CAB 3 mg/week by progressively increasing the CAB dose as needed and tolerated, every 3 months, up to 9 mg/week. Overall, normalization of PRL levels was achieved in 18 patients (72%), as follows: in 3 (12%) patients, with up to 4 mg/week; in 9 (36%) patients, with 5 mg/week; and in 6 (24%) patients, with 6-7 mg/week. No patients benefitted from doses > 7 mg/week. CAB was well tolerated, and no significant echocardiographic valve abnormalities were detected (101).

#### Debulking surgery

Patients can always undergo transsphenoidal surgery if their tumor is potentially resectable and an experienced neurosurgeon is available (4,90). There is also some evidence that debulking surgery may improve the response to DA (6,102–104). Of 61 patients resistant to either BCR or CAB, Hamilton and cols. (102) reported that surgery resulted in a normalization of PRL in 36% without dopamine agonist medication and in 15% with medication. Similarly, Primeau and cols. (102) found in 26 patients resistant to BCR, quinagolide or CAB that surgery resulted in a normalization of PRL in 42% without medication and in 27% with medication. In the European Multicenter Study, the rate of postoperative normalization of PRL was only 7.8% without medication and 5.3% with medication (104).

#### Radiotherapy

Among the functioning pituitary tumors, prolactinomas seem to be the less responsive to radiotherapy (see below). Indeed, although it can also be effective in controlling tumor growth, its efficacy in restoring PRL levels to normal is limited (4,89,90).

#### Other drugs

Of note, if one is dealing with microprolactinomas resistant to DAs, hormonal replacement therapy is often all that is necessary for men and premenopausal women not willing to conceive (4,19,90).

Estrogens may cause a decrease in the effectiveness of dopamine agonists through multiple mechanisms, including direct effects on PRL gene transcription (105), stimulation of mitotic activity (106), and a decrease in the number of D^2^ receptors on the lactotroph cell membrane (107). Moreover, estrogen may block apoptosis (108). Thus, reducing endogenous estrogens in resistant prolactinomas through the use of SERMs in women or aromatase inhibitors in men might be an interesting experimental approach. Accordingly, a few patients with “bromocriptine resistant” invasive macroprolactinomas have been shown to respond with a decrease in tumor size and a lowering of PRL levels when tamoxifen was added (90,109). The use of the SERM clomiphene was also shown to be effective in the recovery of hypogonadism in prolactinoma patients who persisted with low testosterone levels despite maximal doses of DA (110,111). In addition, the successful use of anastrozole, an aromatase inhibitor, was recently reported in two males with DA-resistant prolactinomas (90,112).

Somatostatin analogs generally are not useful for PRL-secreting tumors (90), and there have been few reports on the successful combination therapy of octreotide (113) or lanreotide (114) and cabergoline in cases of DA-resistant tumors. The somatostatin receptor subtype 5 (SSTR5) is the most important with respect to the regulation of PRL secretion (115). Therefore, as pasireotide only has substantial activity at SSTR5 (116), it would be potentially more useful than the SSTR2-agonists octreotide and lanreotide in patients with aggressive DA-resistant prolactinomas (90).

Temozolomide, an oral alkylating agent, has been successfully used in the treatment of aggressive or malignant pituitary tumors since 2006 (117,118). It has also been found to be moderately successful in some very aggressive, large DA-resistant prolactinomas. Reviewing such cases, Whitelaw and cols. (119) found that 12 of 20 (75%) resistant PRL-secreting macroadenomas responded to temozolomide. Given the toxicity of the drug, its use is generally regarded as the therapy of last resort and should only be performed after the failure of DAs, surgery and radiotherapy for tumor control (90,119). Unfortunately, many of these very aggressive tumors escape from the suppressive effects of temozolomide after 0.5-2.5 years (90,119).

Other treatment strategies undergoing clinical trials are the use of chimeric molecules (somatostatin analogues and dopamine D^2^ receptors) (2,3), PRL receptor antagonists (3), and antiblastic drugs, such as mTOR and tyrosine kinase inhibitors (3,120).

**COMMENT 7:** Patients with resistance to bromocriptine should be switched to treatment with cabergoline (CAB), which leads to prolactin normalization in at least 50% of cases. Facedwith resistance to CAB, a stepwise increase in the dose should be performed first, as long as there is a continued response and no adverse effects. This approach is successful in at least 70% of patients. In our experience, doses > 7 mg/week do not provide additional benefits. Debulking surgery may improve a response to the dopamine agonist (DA) when the medical treatment has failed. Radiation and temozolomide can be indicated to control aggressive tumor growth even under DA therapy.

### 2.2 Are there differences in the safety profile of DAs?

DA therapy often precipitates a broad spectrum of side effects, more frequently gastrointestinal symptoms (e.g., constipation, nausea, vomiting, etc.) but also postural hypotension, dizziness and headaches, which may range from mild to severe (34,88,121). The most common adverse events include nausea or vomiting (~35%), headache (~30%), and dizziness or vertigo (~25%) (121). The safety profile of CAB is similar to that reported for BRC, but CAB adverse effects are generally less frequent, less severe, and of shorter duration (34,88), and they resolve with dose reduction or continued use in many patients (34,88,121). The rate of treatment discontinuation due to complete intolerance is also significantly higher with BCR, compared to CAB (~12% vs ~4%) (87).

Whether CAB is associated with an increased risk of clinically relevant cardiac valvulopathy in patients with prolactinomas as in those with Parkinson's disease is still debated (see below) (10,121).

### 2.3 Do dopamine agonists cause psychiatric disorders?

Dopamine antagonists (e.g., haloperidol, olanzapine, quetiapine, sulpiride) are classically used for the treatment of psychiatric disorders such as psychosis and schizophrenia due to the supposed dopaminergic hyperactivity that occurs in these cases (43,46,47). DA treatment exerts the opposite effect to that of the dopamine antagonists (7). These agents can rarely cause psychiatric adverse side effects, including depression, somnolence, anorexia, anxiety, insomnia, impaired concentration, nervousness, hallucinations, nightmares, psychosis, impulsive control disorders (ICDs), and mania (3,5,122–125). The exact incidence of these side effects in DA-treated patients is not known but is thought to range from less than 1% to 3% (122,123).

ICDs are characterized by difficulties in resisting urges to engage in behaviors that are excessive and/or ultimately harmful to oneself or others and can include gambling, kleptomania, intermittent explosive disorder, compulsive sexual behavior and compulsive buying (126).

Few reports have been published focusing on ICDs and other psychiatric disorders in prolactinoma patients treated with CAB (123–133). These reports have described disorders such as a first episode of mania (123,129); mania with psychotic features in a subject with bipolar disorder after starting CAB (125); psychosis in a patient with undiagnosed depression (126); compulsive gambling one year after starting CAB (130); gambling and compulsive sexual behavior just after starting CAB (131); psychosis in a patient without a previous history of psychiatric disorder three months after starting CAB (132); and depression and compulsive buying (133).

In 2011, Martinkova and cols. (128) published a review reporting a 10% prevalence of ICD in patients on CAB for prolactinoma treatment. In another study, males with prolactinomas treated with DAs have been reported to be 9.9 times more likely to develop an impulse control disorder compared to their counterparts with nonfunctioning pituitary adenomas (134). More recently, Barake and cols. (135) conducted the only randomized study aiming to assess the impact of CAB on the appearance of ICDs and concluded that patients on CAB (particularly with higher dosages) were more susceptible to impulsivity disorders than those not taking the drug.

Psychiatric effects of CAB can be modulated by P-glycoprotein (P-gp). This protein, encoded by the *ABCB1* gene (136), is a membrane transporter expressed in tumor cells and in normal tissues that acts as an efflux transporter in order to transport certain substances out of the brain and protect it against harmful substances. A recent study showed that CAB is a P-gp substrate (137). This study demonstrated that *ABCB1* gene polymorphisms (with loss of function) could explain individual differences in the occurrence of central nervous system side-effects, demonstrating a future perspective to select patients for CAB treatment based on this gene study (137).

**COMMENT 8:** In susceptible subjects, psychotic symptoms and impulsive control disorders may be triggered or aggravated by dopamine agonist (DA) therapy. It seems therefore important to exclude psychiatric disorders before prescribing DAs and to carefully follow up patients prone to or with a history of psychiatric disorders.

### 2.4 Risk of heart valvular disease associated with dopamine agonists – what is its relevance?

The main warning signs that cause concerns among endocrinologists about a possible association between the therapeutic use of dopamine agonists (DAs) with heart valvular disease (HVD) emerged from two articles published in 2007 involving patients with Parkinson's disease (138,139). In this group of patients, there is now strong evidence that treatment with high doses of CAB and pergolide (PGL) is associated with a higher risk of cardiac valvular regurgitation and moderate evidence that treatment with CAB, and the non-ergot-derived DAs pramipexole is associated with a higher risk of heart failure (140). The underlying mechanism involves the activation of the 5-hydroxytryptamine 2B (5-HT2B) receptors, abundantly expressed on heart valves, which induces a cascade of events involving mitogenesis, fibroblast proliferation, valve thickening and, finally, valvular dysfunction (141,142). DAs bind to 5-HT2B receptors with different affinities; for instance, CAG and PGL show high affinity and act as a potent agonist, whereas bromocriptine (BRC) has a lower affinity compared to other DAs (141,142). However, there have been case reports and clinical studies in Parkinson's disease reporting on an increased risk of abnormal valvular regurgitation and cardiac and non-cardiac fibrotic reactions in patients treated with BRC (143,144).

As expected, the findings in patients with Parkinson's disease brought an immediate concern about the risk of HVD in endocrine patients treated with DAs, especially CAB, despite the striking differences between these groups that must be highlighted. First, the daily and cumulative doses of DAs used in neurology (at least 3 mg of CAB per day) are approximately 10-fold higher than those used in endocrinology (rarely more than 3 mg of CAB per week) (145). Moreover, most studies in Parkinson's disease predominantly include men older than 60 years of agewho are usually on therapy with multiple drugs, while patients with endocrine abnormalities treated with DAs are predominantly young or middle-age women (146). This is important because the prevalence of valvular regurgitation increases with age and is influenced by gender, body mass index and hypertension (146,147). Another factor to be considered is the impact of examiner bias in the results, as shown in the study by Gu and cols. (148).

In patients with hyperprolactinemia, most cross-sectional studies were dedicated to investigating the role of CAB in HVD. A meta-analysis of seven observational studies with 1,398 individuals found an increased risk of mild-to-moderate tricuspid valve regurgitation in hyperprolactinemic patients taking CAB. No significant differences were observed in mitral or aortic valve regurgitation (149). Another recent systematic review analyzed data from 21 studies and from 40 patients followed by the authors (150). Utilizing the precise definition of CAB-associated valvulopathy as the triad of moderate or severe regurgitation associated with a restricted and thickened valve, clinically relevant disease was identified in only two out of 1,811 patients, resulting in a prevalence of 0.11% (150). The authors of this study also prospectively assessed 40 patients with prolactinoma taking cabergoline. Cardiovascular examination before echocardiography detected an audible systolic murmur in 10% of cases (all were functional murmurs), and no clinically significant valvular lesion was shown on echocardiogram in the 90% of patients without a murmur (150).

A Brazilian study by Boguszewski and cols. (151) was the first to include a group of prolactinoma patients treated with BRC for comparison. Notably, a higher prevalence of trace tricuspid regurgitation was observed in patients taking BRC, while more prevalent trace-to-mild tricuspid and trace mitral regurgitation and a higher mitral tent area were observed in CAB users; nevertheless, all these echocardiographic findings were not clinically significant (151). This observation was confirmed in a subsequent study in which an increased prevalence of subclinical HVD in patients with prolactinomas on long-term treatment with either BRC or CAB was reported (152).

Beyond the cross-sectional investigations, longitudinal studies have shed light on the potential damage caused by DAs to cardiac valves. Delgado and cols. (153) followed patients with prolactinomas treated (*n* = 45) or not treated (*n* = 29) with CAB for 2 years and found an increased prevalence of valvular calcification in CAB users that was not accompanied by a higher prevalence of valvular dysfunction. Auriemma and cols. (154) concluded that 5 years of CAB therapy in prolactinomas does not increase the risk of significant cardiac valve regurgitation. Similarly, 19 patients with prolactinomas were followed in Curitiba, Brazil, during 5 years of continuous treatment with DAs. Neither a significant change in valvular regurgitation grades nor symptomatic disease was observed in the follow-up (155). Population-based studies have also confirmed the safety of CAB in endocrine patients. A nationwide cohort study carried out in Denmark did not support that hyperprolactinemia or its treatment is associated with an increased risk of clinically significant HVD (156). In a multi-country, nested case-control study involving data from the United Kingdom, Italy, and the Netherlands, CAB was associated with an increased risk of HVD in Parkinson's disease but not in hyperprolactinemia (157), confirming the role of high cumulative doses in the development of valvular dysfunction and the safety of long-term use of low cumulative doses.

In two studies involving 51 prolactinoma patients considered to be resistant to standard doses of CAB, no significant echocardiographic valve abnormalities were detected despite the use of doses ranging from 3 to 12 mg/week (99,110).

The absence of a detrimental effect of CAB therapy on cardiac valves has also been noticed in a longitudinal study in patients with acromegaly (158).

The findings of the abovementioned studies challenge the US Food and Drug Administration (FDA) advice that patients with prolactinoma treated with CAB should have an annual echocardiogram to screen for valvular heart disease. Of note, with conventional doses of cabergoline, i.e., up to 2 mg/week, usually used in patients with prolactinomas, there does not appear to be any increased risk of cardiac valve abnormalities (90,145,156). However, as it is uncertain at what dose level these valve effects become significant, some experts recommend assessing all patients receiving > 2 mg/week with an echocardiogram on a yearly basis (90). More recently, based on the findings of their systematic review, Caputo and cols. (150) proposed that such patients should be screened by a clinical cardiovascular examination and that echocardiograms should be reserved for patients with an audible murmur, those treated for more than 5 years at a dose of more than 3 mg per week, or those who maintain cabergoline treatment after the age of 50 years. Nevertheless, special attention should be given in interpreting echocardiographic findings in older individuals, as highlighted by some authors (146,147).

Subclinical valvular abnormalities detected by echocardiography are not an indication for cessation of the therapy (121,154). Importantly, if valve lesions are detected or progress during follow-up, further evaluation is indicated to distinguish CAB-induced etiologies from other causes of valvulopathies. In such cases, therapy may be interrupted, reduced or maintained based on clinical judgment and discussion with the patient (121,150,154).

**COMMENT 9:** Current available data do not support major concerns about the risk of valvulopathy in patients with hyperprolactinemia or other endocrine diseases who are chronically treated with DAs in standard doses. Thus, an echocardiogram evaluation would not be recommended for patients receiving cabergoline at doses up to 2 mg/week.

### 2.5 Withdrawal of the dopamine agonist – Why, when, how and how often?

#### Why, when and how?

Although it is well known that prolactinomas respond very well to DAs, the optimal duration of treatment is still not clear (4,6,10). Despite the need for long-term therapy, withdrawal of treatment should be considered because of the adverse effects of medical treatment, potential long-term consequences in cardiac valves, and treatment costs (4,6,88).

Studies on persistent normoprolactinemia after DA withdrawal have been published since 1979 (159). In 2002, a Brazilian study by Passos and cols. (160) showed a remission rate of 20.6% after BCR discontinuation. In 2003, a landmark prospective study by Colao and cols. (161) demonstrated that CAB could be successfully withdrawn in 70% of patients with a MIC and 64% of those harboring a MAC (161). An extension of this study to 8 years documented remission rates of 66 and 47%, respectively (162). The higher success rates of this study were attributed to stricter selection criteria for CAB withdrawal, including a prolonged period of normoprolactinemia during treatment and a significant tumor size reduction (161,162). Since 2003, several studies have evaluated the recurrence rates of hyperprolactinemia after DA withdrawal with variable results (162–170).

In a meta-analysis of 19 studies with a total of 743 patients, the overall remission rate after the withdrawal of DA therapy was only 21%, 32% for idiopathic hyperprolactinemia, 21% for microadenoma and 16% for macroprolactinomas (171). Furthermore, a higher rate of remission was found in studies in which CAB was used (35% in 4 studies) versus BRC (20% in 12 studies) (p = 0.07) (171). In studies lasting over 24 months of treatment, the remission was higher (34%) in comparison to studies with a shorter treatment duration (16%) (p = 0.01) (153). The remission rate was also higher in studies in which agreater than 50% reduction of the tumor was achieved in all patients prior to discontinuation of therapy (171).

The guidelines provided by the Endocrine Society in 2011 recommended that patients who have attained normoprolactinemia for at least 2 years and who have no visible tumor remnant on MRI may be candidates for a gradual DA withdrawal (4).

A meta-analysis from 2015 that only evaluated patients submitted to CAB withdrawal found that the hyperprolactinemia recurrence rate was 65% by a random effects meta-analysis (172). In a random effects meta-regression adjusting for optimal withdrawal strategies, a CAB dose reduced to the lowest level before withdrawal was associated with treatment success (p = 0.006), whereas CAB treatment longer than 2 years showed no trend of effect (p = 0.587). Patients who received the lowest CAB dose and presented a significant reduction in tumor size before withdrawal were more likely to benefit from CAB withdrawal (p < 0.001) (172).

In three recent studies, the recurrence rates after CAB withdrawal ranged from 27% to 54% (168–170). Among 74 patients (19 MACs and 55 MICs) treated with CAB for ≥ 3 years, recurrences occurred within 12 months in 34 (45.9%), regardless of the previous duration of CAB therapy (up to 3 yrs, 3 to 5 yrs or > 5 yrs) or initial adenoma size (168). Among 67 patients (23 MACs and 44 MICs), the overall recurrence rate was 54% (35% in cases of MIC and 64% in subjects with MAC) (169). A higher remission rate was found with CAB, compared to BCR (55% vs. 36%) (169). More recently, treatment discontinuation was evaluated in 11 patients with macroprolactinomas treated with CAB for at least 5 years (170). Recurrences of hyperprolactinemia were observed in 3 (27%) post-withdrawal patients at a median time of 3.0 (range; 2.9-11.2) months, indicating that a high percentage (73%) maintained remission for at least 12 months after CAB cessation (170).

#### How often?

Two independent studies (173,174) have investigated the outcome of a second attempt at CAB withdrawal in patients with recurrence of hyperprolactinemia after the first withdrawal, receiving additional CAB therapy for at least 2 years. The results of these studies have demonstrated that a second attempt at CAB withdrawal after 2 additional years of therapy may be successful in approximately 30% of patients (173,174).

#### What are the predictors of remission?

Several studies in the literature sought the predictors of remission after stopping DAs, but the results of these studies have been contradictory (4,6,10,19). Overall, parameters more often associated with a successful DA withdrawal include suppressed PRL levels, the use of low doses of CAB, and lack of a visible tumor on MRI before withdrawal. However, hyperprolactinemia recurrence was observed even in patients who presented with these parameters (168–172).

In the great majority of studies, patients harboring MACs presented a lower risk of hyperprolactinemia remission than those with MICs when treatment is discontinued (161–169). The same was true when CAB and BCR were compared (167–169). In the series by Dogansen and cols. (169), the overall remission rate was 46% (65% in cases of MIC and 36% in subjects with MAC). A higher remission rate was experienced by patients treated with CAB vs. BCR, for both MIC (86% vs. 56%) and MAC (45% vs. 27%), respectively (169).

Menopause might be considered a factor that influences the reduction of PRL levels (175). Nevertheless, Colao and cols. (163) showed that the rate of recurrence of hyperprolactinemia was similar among premenopausal and postmenopausal patients.

There are few studies showing that some patients may spontaneously normalize serum PRL concentrations after pregnancy. Auriemma and cols. (176) reported that pregnancy was associated with normalization of PRL levels in 68% of patients. Moreover, breastfeeding did not increase the recurrence rate of hyperprolactinemia (177). Thus, DA withdrawal has been suggested after pregnancy to assess the possibility of hyperprolactinemia remission (176,177). Previous pituitary surgery and radiotherapy also favor persistent normal PRL levels after DA withdrawal (4,6,88).

#### How long should patients be followedup after withdrawal?

The follow-up after DA withdrawal has varied in different studies. Generally, the patients have been observed for 1 to 5 years after cessation of treatment (171,172). Most patients relapse within the first year, during which the patient should be subjected to a stricter monitoring of PRL levels (e.g., every 3 months) (164–169). In virtually all studies, all recurrences were observed within 24 months of DA discontinuation (162–173). In cases of hyperprolactinemia recurrence, the risk of tumor growth is very low (< 10%) (88,160,162,169).

**COMMENT 10:** Following two years of treatment, cabergoline (CAB) withdrawal should be strongly considered in all patients with microprolactinomas and those with macroprolactinomas without visible tumor or harboring small tumor remnants, in the presence of PRL levels < 10 ng/mL and the use of the lowest dose of CAB. Before drug withdrawal, it is recommended to gradually taper the CAB dose.

### 2.6 What is the role of surgery for prolactinomas?

Transsphenoidal surgery (TSS) represents the golden standard of surgical approach for both microprolactinomas and most macroprolactinomas (88), whereas craniotomy must be reserved for tumors inaccessible via the transsphenoidal approach and currently is indicated in extremely rare cases (6,88,178).

Additional indications for TSS surgery are complications of the prolactinomas, particularly the management of symptomatic apoplexy (178) and the surgical repair of CSF leaks (179). The latter can occur spontaneously due to tumor invasion of the sphenoid sinus or result from a rapid tumor shrinkage induced by DA therapy (180–182). In a recent review, more than 90% of cases of cerebrospinal fluid leakage were related to the use of DA, with a mean time of 3.3 months between the start of drug administration and the diagnosis of rhinorrhea (182). However, this complication can also occur during long-term treatment (183). An additional strategy to CSF leakage would be the withdrawal of DA to allow tumor re-growth to stop the leak (181). As β2-transferrin is only found in CSF, its detection in nasal secretions is a very accurate tool for confirming the diagnosis of CSF rhinorrhea (179,184).[Table t5]

**Table 5 t5:** Indications for surgery in prolactinomas

Increasing tumor size despite optimal medical therapy
Pituitary apoplexy
Intolerance to dopamine agonists
Resistance to dopamine-agonists
Persistent optic chiasm compression
Cerebrospinal fluid leak during administration of dopamine agonists

Adapted from Refs. 6, 10, and 19.

#### Efficacy

The most important determinants of successful surgery for prolactinoma treatment are the experience of the neurosurgeon, moderately increased serum PRL levels (< 200 ng/mL), and tumor size and invasiveness (6,121). In a literature review involving more than 50 series, initial surgical remission, defined as the normalization of PRL levels, occurred on average in 74.7% and 34% of patients with microprolactinomas (MICs) and macroprolactinomas (MACs), with a recurrence rate of 18% and 23%, respectively (88). More recently, the analysis of surgical results from 13 published series, including at least 100 patients, has shown the control of PRL levels to be achieved in approximately 73% of 1211 microprolactinomas and 38% of 1480 macroprolactinomas (185).

Notably, as shown in two recent studies (102,104), many patients with partial resistance to CAB achieve PRL normalization after surgical debulking, using a lower dose of CAB than the presurgical one.

#### Safety

Complications from TSS for MIC are infrequent, the mortality rate being at most 0.6% and the major morbidity rate being approximately 3.4% (88,121). In patients with large tumors, particularly those with giant prolactinomas, the mortality rate ranges from 3.3% to 31.2% (88,121). Visual loss, stroke/vascular injury, meningitis/abscess, oculomotor palsy and cerebrospinal fluid rhinorrhea have been reported to occur in 0.1%, 0.2%, 0.1%, 0.1% and 1.9% of cases, respectively (121,186–188). Transient diabetes insipidus (DI) is quite common with TSS for both micro- and macroadenomas (121). In contrast, permanent DI is rare and has been reported to occur in approximately 1% of surgeries on MACs (121,187,188). Hypopituitarism, commonly found in patients with macroadenomas before surgery as a consequence of mass effects, has been reported either to further worsen or to improve after surgery (102–104).

**COMMENT 11:** Surgery, usually by the transsphenoidal approach, is indicated for patients with resistance or intolerance to dopamine agonists; macroprolactinomas with chiasmal compression and visual impairment without fast improvement by medical treatment; and acute tumor complications, such as symptomatic apoplexy or cerebrospinal fluid leakage.

### 2.7 What is the role of radiotherapy for prolactinomas?

As prolactinomas are among the most radioresistant pituitary tumors, PRL normalization occurs in only approximately one-third of patients submitted to radiotherapy (RT) (88,189,190). Moreover, it is associated with a high risk of radiation-induced hypopituitarism at 10-20 years (121,191). Therefore, RT is only indicated to control tumor growth in DA-resistant cases not controlled by surgery, as well as in the very rare cases of malignant prolactinomas (4,6,88).

Historically, patients have been treated with both external beam radiotherapy and stereotactic techniques (189). External beam radiotherapy, also referred to as “conventional radiotherapy” (CRT), involves the use of multiple non-overlapping beams of X-rays that intersect over the target area (191). Stereotactic RT is a form of RT that uses stereotactic guidance to deliver high doses of radiation in a single fraction (radiosurgery) or in multiple fractions (191).

#### Efficacy

A comprehensive review of the literature regarding hormonal normalization in patients with prolactinomas treated with CRT or radiosurgery concluded that the overall normalization rate of both approaches seems to be similar (34.1% for CRT vs. 31.4% for radiosurgery) (88). In the series by Wan and cols. (190), of the 176 patients with prolactinomas submitted to gamma-knife radiosurgery, 23.3% achieved PRL normalization, whereas in 90.3%, the tumor volume decreased or remained unchanged.

Stereotactic techniques deliver less radiation to normal brain and nerve tissue in the tumor vicinity. Thus, radiation-induced complications are thought to be less frequent with radiosurgery than with CRT (88,189,190). Moreover, a shorter time to hormonal normalization with radiosurgery has been reported in some series (189,191), but not in all (192).

Currently, radiosurgery has been the recommended treatment option unless the tumor is larger than 3-4 cm, or within 3 mm of the optic nerves, chiasm or tracts (192). In this situation, patients should be offered CRT or, whenever possible, stereotactic RT in multiple fractions (88,189–191).

#### Safety

The use of convention radiotherapy (CRT) is associated with the development of several severe complications (121). More than 50% of patients receiving pituitary radiotherapy will develop at least one anterior pituitary hormone deficiency within the following decade (193,194). Although hypopituitarism tends to arise in the first 5 years after radiation treatment, new deficiencies can appear even 20 years later (103,176,177). Additional complications of CRT include cerebrovascular accidents (CVAs), a second brain tumor and optic nerve injury. The incidence of CVA has been found to increase from the time of radiation, from 4% at 5 years to 11% at 10 years and 21% at 20 years (121,195). The cumulative risk of second brain tumors has been demonstrated to range from 2.0% at 10 yr, to 2.4% at 20 yr, and 8.5% at 30 yr (196). The rate of presumed radiation-induced optic neuropathy has been estimated to be 0.8% at 10 years (197). Another rare complication is radiation-induced necrosis of the surrounding brain tissue, with a prevalence of approximately 0.2-0.8% (88,121).

In patients receiving radiosurgery, hypopituitarism has been found to be the most common complication, occurring in approximately one-third of patients (121,198). Clinical deterioration due to visual field loss and worsened facial sensory loss, as well as a handful of cases of second brain malignancies have also been reported (88,191,199).

These data suggest the greater safety of radiosurgery compared to CRT. However, further studies of large series and with long follow-ups are required to draw definitive conclusions (121).

**COMMENT 12:** Due to its low efficacy and potentially severe side-effects, radiation therapy is only indicated to control tumor growth in DA-resistant cases not controlled by surgery. Whenever possible, preference should be given to stereotactic techniques.

### 2.8 How giant prolactinomas should be managed?

Although there is no consensus on the definition of giant prolactinomas (GPs), they are usually defined as tumors with a maximum diameter greater than or equal to 4 cm (200–202).

GPs represent only 2-3% of all prolactinomas, and they are more commonly found in middle-aged men (200,201). These tumors are usually more aggressive in men than in women (203). GPs often cause visual field defects and/or ophthalmoplegia due to compression of the optic chiasm and/or cranial nerves, respectively, and headaches, as well as other neurological alterations and other atypical manifestations for a pituitary adenoma, such as orbital invasion, epistaxis or obstructive hydrocephalus (204–207). Panhypopituitarism may also be present. Therefore, formal visual testing is usually necessary, and pituitary function evaluation is mandatory (200,203).

Different therapeutic approaches, such as dopamine agonists (DAs) alone or in combination with surgery and radiotherapy, may be necessary to reach the therapeutic goals in patients with GPs, which include tumor volume control, normalization of the PRL level and restoration of eugonadism (200). Temozolomide (TMZ) has been used in aggressive prolactinomas that are unresponsive to the abovementioned therapies (117–119). Due to the large tumor volume and commonly invasive behavior, control of mass effects should be a priority in the management of patients with giant prolactinomas (88).

#### Dopamine agonists

As medical therapy in most cases causes a marked and rapid reduction of the tumor size, it is considered the first-line treatment option in patients with GPs, even in the presence of visual abnormalities. However, close monitoring of the visual field is mandatory (200–202). In this setting, early surgery (e.g., within 15 to 30 days) may occasionally be necessary if the tumors do not shrink and if severe visual field defects do not improve or even get worse (200–202).

The use of DA for GPs seems to reduce significantly the tumor mass (> 30% decrease in tumor diameter or > 65% reduction in tumor volume) in 74% of the cases and to normalize the PRL level in 60% of the cases (200). Cabergoline (CAB) is the preferred DA for the medical management of GPs due to its greater efficacy and better tolerability compared to BCR (34,88). In the series by Espinosa and cols. (208), CAB treatment resulted in the normalization of PRL levels in 68% and in the reduction of > 50% in tumor volume in 87% of the GP patients. The composite goal of PRL normalization and > 50% tumor reduction was achieved by 55% of patients with GPs (n = 26) and by 66% of patients with no giant macroprolactinomas (n = 100) (p = 0.19) (208). Among GPs ≥ 6 cm, DA treatment achieved PRL normalization in 11/18 (61%) patients within a median interval of 20 months (209). In the series by Vilar and cols. (210), 10 of 16 patients (62.5%) reached PRL normalization at weekly doses ranging from 2.5 to 7 mg. Regarding DA withdrawal, there are no data in the literature about CAB withdrawal in patients with GPs. Concerning BRC withdrawn in these patients, some authors described the persistence of normoprolactinemia and tumor volume shrinkage in only a minority of patients (211–213).

#### Surgery

Neurosurgery should be considered as part of the multidisciplinary approach to patients with DA-resistant prolactinoma (debulking surgery) or should be restricted to some acute complications such as apoplexy or leakage of cerebrospinal fluid during DA therapy (4,206,214). Neurosurgery could also be considered for patients with severe visual field defects that do not show any improvement or even worsen during DA treatment. The transsphenoidal approach is usually the first choice, even for GPs (200). In patients with giant and invasive prolactinomas, surgery is hardly curative, regardless of the surgical technique employed or the neurosurgeon's experience. Therefore, in these cases, the goal of surgery is to debulk the tumor in order to improve the symptoms related to mass effects (88,121). Debulking surgery may also improve the tumor response to DA therapy (102,104).

#### Radiotherapy

Radiotherapy should be used in patients with GPs who do not achieve disease control with DA therapy and surgery, particularly regarding mass effects (200–202).

#### Temozolomide

Temozolomide is an oral alkylating chemotherapeutic agent that exerts cytotoxic effects through methylation of DNA and can be used in aggressive giant prolactinomas that remain uncontrolled in terms of mass effects despite the multiple treatment modalities previously mentioned. The standard dose is 150–200 mg/m^2^ for 5 days, repeated every 28-days in a cycle (117,119).

**COMMENT 13:** Cabergoline is the therapy of choice for patients with giant prolactinomas (GPs), as this drug enables normalization of prolactin levels and significant tumor reduction in the majority of cases, but higher doses are often required, compared to those for smaller macroprolactinomas. Surgery should be considered as part of the management of these patients, especially in patients who still have a large tumor load despite dopamine agonist therapy. Radiotherapy and/or temozolomide should be reserved for the subgroup of GPs that are not controlled in terms of mass effects regardless of the multiple conventional treatment modalities.

### 2.9 How to manage the challenges involving prolactinomas and pregnancy?

Chronic anovulation and infertility are very frequent among women with prolactinomas, mostly due to the hypogonadotropic hypogonadism related to hyperprolactinemia (6,10,88). Hyperprolactinemia decreases the luteinizing hormone (LH) pulse amplitude and frequency through the suppression of GnRH (14). This effect appears to be mediated by an earlier step of suppressing the generation of kisspeptin, a protein made by neurons in the arcuate and periventricular nuclei of the hypothalamus, which stimulates GnRH release (215). PRL can also decrease estrogen and progesterone production through direct effects on the ovaries (216). Fertility is, however, restored in most women with the use of DA. In the absence of hormonal control in cases with microprolactinomas, clomiphene citrate or recombinant gonadotropins may be used for ovulation induction (216,217).

During pregnancy, the primary concern is growth of the tumor because of high levels of estrogens, leading to visual disturbances and headache. The second point of concern is the fetal exposure to DA in early embryogenesis and the potential risk of malformations (216,217).

#### Tumor growth

Prolactinomas can enlarge during pregnancy as a result of both the stimulatory effect of these high estrogen levels and the discontinuation of the dopamine agonist that might have caused tumor shrinkage (216). In microprolactinomas, the chance of clinically significant tumor growth is less than 5%; therefore, after pregnancy confirmation, DA can be withdrawn, and the patient should be monitored clinically every trimester. In contrast, in patients with macroadenomas, the risk of tumor growth with clinical repercussion is up to 35% (217). However, Holmgren and cols. (218) noted a reduced risk of tumor growth during pregnancy in patients treated with BRC for at least 12 months. Thus, in patients with expansive macroprolactinomas, it is mandatory to observe a tumor within the sellar boundaries and usually to wait at least 1 year under treatment with a DA (217,219).

#### DAs safety in the fetus

BRC has been shown to cross the placenta in human studies (103); CAB has been shown to do so in animal models, but such data are lacking in humans (121,216). As a general approach in pregnancy, DAsare discontinued as soon as pregnancy is confirmed (216). Nevertheless, taking into account that CAB has a long half-life and can be detected in the circulation within 30 days after drug withdrawal, early fetal exposure is unavoidable (217). Moreover, the experience with this drug is still limited compared to BRC: ~950 vs. ~6200 pregnancies induced by CAB and BRC, respectively (216). These facts are the rational for BRC preference in hyperprolactinemic women willing to become pregnant (217), as recommended by the Guidelines of the Endocrine Society, with BRC being the only authorized DA for pregnancy induction (4). However, in clinical practice, CAB has often been used for this purpose with no apparent damage to the fetus (216).

The potential fetal malformation and impairment of the dopaminergic brain circuitry under DA use are a concern (216,217,219). Addressing this issue, Molitch (216) has compared outcomes data from 6239 pregnancies induced by BCR with 968 pregnancies induced by CAB and pregnancies in the US general population, regarding miscarriages, births at term, premature births, multiple pregnancies, and malformed newborns. Overall, no difference was found among the three groups (216).

In the vast majority of cases, the DA was withdrawn at up to 6 weeks of pregnancy. In the literature, there are only data on approximately 100 cases of BRC use throughout pregnancy, which have disclosed one case of undescended testis and one case of talipes deformity (217,220,221). Recently, a compilation of 15 pregnancies with the use of CAB during the entire pregnancy has shown 12 healthy babies and one fetal death by severe pre-eclampsia (222).

Concerning the long follow-up of children conceived during BRC treatment, Bronstein followed up 70 children, during 12 to 240 months, and there was one case of idiopathic hydrocephalus, one child with tuberous sclerosis and another one with precocious puberty (223). In two other studies (217,224), no impairment of physical development was observed.

In four studies, the follow-ups of children conceived on CAB were described. Bronstein (223) and Ono and cols. (225) did not find any abnormalities in five and 83 children followed for 41 months and 12 years, respectively. Lebbe and cols. (226) following 88 children described two cases of slight delay in verbal fluency and one case of difficulty in achieving complete continence. Moreover, Stalldecker and cols. (227) followed 61 children and found two cases of seizures and two cases of pervasive developmental disorder.

In summary, BCR and CAB seem to be equally safe for the fetus; none of these drugs are apparently associated with increased risk for abortions or malformations, when used for pregnancy induction in infertile hyperprolactinemic women. Ideally, CAB should be discontinued 30 days before the conception (216,218,223).

#### The best approach

**Figure 8:**
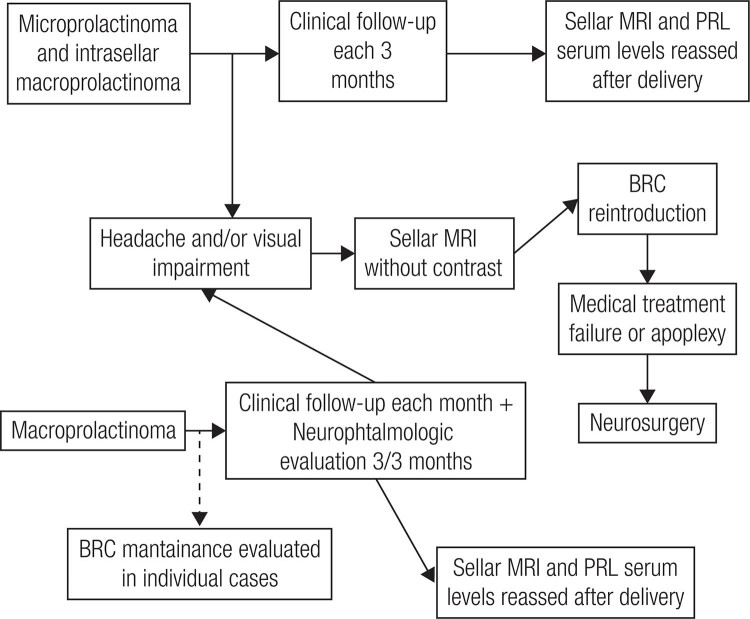
Algorithm suggested for the prolactinoma management during pregnancy (PRL: prolactin; BCR: bromocriptine; MRI: magnetic resonance imaging) (Adapted from Ref. 217).

An algorithm proposed by Glezer and cols. (217) for the management of prolactinoma during pregnancy is shown is Figure 8. The DA should be withdrawn as soon as pregnancy is confirmed in patients with microadenomas or enclosed macroadenomas. Assessment of tumor shrinkage in macroadenomas is mandatory before pregnancy allowance. In previously expanded or invasive tumors, the choice of BRC maintenance throughout pregnancy or its reintroduction if needed, or else the indication of previous surgical debulking, depends on the physician's and patient's decision (217).

Patients with microprolactinomas (MICs) or enclosed macroprolactinomas (MACs) should be followed each trimester, with attention to headache and visual impairment (217). In the presence of those complaints, sellar MRI without contrast, preferably after the first trimester, should be performed, and BRC reintroduction is indicated if tumor growth is related to the clinical findings (217). In cases of BCR intolerance, CAB, although off-label, could be used instead (217,219,222). On the other hand, in patients with expanding MACs, DA maintenance throughout pregnancy should be evaluated by an expert (217,219). Clinical evaluation monthly and neuroophthalmological evaluation each trimester are indicated. In patients with MICs or MACs experiencing symptoms related to mass effects and without improvement with clinical treatment, neurosurgery should be performed, preferably during the second trimester (216,217). An alternative approach might be delivery of the baby, if the pregnancy is sufficiently advanced (e.g., > 37 weeks) (216,217,219).

Notably, during pregnancy, periodic checking of PRL levels is of no diagnostic benefit and can be misleading (216,217). Indeed, in normal women, PRL levels rise with gestation, but PRL levels do not always rise with tumor enlargement, and tumor enlargement can occur without a change in the PRL concentration (216). A rise in PRL may well not indicate tumor enlargement and therefore may cause unnecessary worry. By contrast, the lack of a rise in PRL may be falsely reassuring in a patient with headaches or other evidence of tumor enlargement (216).

#### Follow-up

After pregnancy, PRL levels and tumor size should be reassessed because reduction, or even complete tumor disappearance, after pregnancy may occur. Moreover, asymptomatic tumor enlargement may occur during pregnancy (216,217). It has been suggested that high levels of estrogen during pregnancy can induce areas of tumor necrosis and apoptosis. The median rate of hyperprolactinemia remission after pregnancy in eight studies was 27%, ranging from 10 to 68% (217).

Breastfeeding does not seem to be associated with tumor growth risk, and it is allowed in patients who did not require DA during pregnancy (216–219).

**COMMENT 14:** In the large majority of cases, a dopamine agonist is only necessary to induce pregnancy and should be discontinued as soon as pregnancy is confirmed. Nevertheless, in selected cases harboring invasive macroprolactinomas or in patients with marked growth and visual and/or headache complaints, the drug may be used throughout pregnancy.

**COMMENT 15:** Bromocriptine and cabergoline seem to be equally safe for the fetus, when used for pregnancy induction. Indeed, short-term exposure to these drugs for less than 6 weeks of gestation has not been found to cause any increase in spontaneous abortions, ectopic pregnancies, trophoblastic disease, multiple pregnancies, or congenital malformations.

### 2.10 How to manage the psychotropic-induced hyperprolactinemia?

It should always be kept in mind that patients with apparent psychotropic-induced hyperprolactinemia may have an alternative etiology for the PRL elevation, such prolactinomas, primary hypothyroidism, macroprolactinemia, or pseudo-prolactinoma (1). Ideally, a baseline PRL level should be obtained before starting psychotropic medications known to increase PRL, but in the majority of cases, this is not practically possible, particularly when these medications are started urgently (4,44). Endocrine Society Guidelines suggest discontinuing the drug for 3 days or substituting with an alternative drug if psychotropic-induced hyperprolactinemia is suspected and then remeasuring PRL (4). Nevertheless, the decision to withdraw or substitute the medication should always be made in consultation with the patient's psychiatrist (4,46). If the drug cannot be safely withdrawn due to the risk of a relapse of psychiatric symptoms and if the onset of hyperprolactinemia does not coincide with the initiation of therapy, then pituitary MRI should be performed to exclude or diagnose a sellar region lesion (4,46).

The different approaches proposed for symptomatic patients with confirmed psychotropic-induced hyperprolactinemia include (i) switching the offending drug to aripiprazole or other atypical antipsychotics (AAPs) with a low effect on PRL levels (41,209–211) and (ii) adding DA therapy (41).

#### Switching to aripiprazole or other AAPs

Because the atypical antipsychotic aripiprazole is a partial D2 receptor agonist, it has a neutral effect on PRL levels and may often decrease them (46, 228). Thus, it may be used as alternative therapy for patients with antipsychotic-induced hyperprolactinemia (46,228). Other atypical antipsychotics, such as quetiapine, olanzapine, lurasidone, asenapine, or clozapine, cause only a mild elevation in PRL levels, and only in a subgroup of those treated (43,46). Therefore, these drugs may also serve as alternatives to antipsychotics with robust effects on PRL (e.g., risperidone) (43,46).

However, in patients who are clinically very stable on antipsychotic treatment, switching to a different antipsychotic such as aripiprazole or other atypical antipsychotics may be inappropriate (46–48). In these cases, using aripiprazole as adjunctive therapy may be useful. Indeed, in a placebo-controlled trial, adjunctive aripiprazole normalized drug-induced hyperprolactinemia in more than 80% of patients with schizophrenia (229). More recently, in a double-blind, randomized, placebo-controlled trial involving patients with risperidone-induced hyperprolactinemia, normalization of PRL levels was encountered in 46% of patients who received adjunctive aripiprazole therapy (49).

Very interestingly, aripiprazole therapy was recently shown to be effective in normalizing PRL levels and in reducing tumor size in two patients with microprolactinomas and psychotic symptoms (230,231).

#### Adding DA therapy

The use of DA in the treatment of antipsychotic-induced hyperprolactinemia is not well studied (46,47). DA and antipsychotic medications may have opposing mechanisms of action. Indeed, the therapeutic effects of most antipsychotics come from D^2^ receptor antagonism (232,233). Thus, there is a concern that DA therapy may reduce the effectiveness of antipsychotic drugs and trigger relapse or exacerbation of psychosis (46). There is, however, little evidence to support this concern. For instance, Chang and cols. (234) reported a cabergoline-induced psychotic exacerbation in three schizophrenic patients when CAB was introduced for the treatment of the hyperprolactinemic symptoms. Likewise, Santos Andrade and cols. (235) described a similar situation when BCR was added for macroprolactinoma treatment in a patient with schizophrenia on haloperidol. By contrast, in 5 studies that involved 174 schizophrenic and bipolar patients and had a duration ranging from 8 weeks to 6 months, the association of dopamine antagonists with low doses of CAB or BCR for the treatment of hyperprolactinemic symptoms apparently did not impair the underlying psychiatric disorder (236–240).

DA therapy should therefore be reserved for patients with severe hyperprolactinemia who fail to respond to aripiprazole as an alternative or adjunctive treatment. Due to its higher specificity for the D^2^ receptor, CAB would be preferable to BCR in the management of antipsychotic-induced hyperprolactinemia. Nevertheless, it should be used cautiously, in doses as low as possible (46).

**COMMENT 16:** The best approach for symptomatic antipsychotic-induced hyperprolactinemia would be the use of aripiprazole as an alternative or adjunctive therapy. DA therapy at low doses can be used cautiously in patients who do not respond to the first approach.
